# Foliar selenium biofortification of soybean: the potential for transformation of mineral selenium into organic forms

**DOI:** 10.3389/fpls.2024.1379877

**Published:** 2024-05-02

**Authors:** Tomáš Mrština, Lukáš Praus, Jiřina Száková, Lukáš Kaplan, Pavel Tlustoš

**Affiliations:** Department of Agroenvironmental Chemistry and Plant Nutrition, Czech University of Life Sciences in Prague, Prague, Czechia

**Keywords:** Glycine max L., sodium selenate, Se recovery, selenium species, selenomethionine, selenocysteine, field experiment

## Abstract

**Introduction:**

Selenium (Se) deficiency, stemming from malnutrition in humans and animals, has the potential to disrupt many vital physiological processes, particularly those reliant on specific selenoproteins. Agronomic biofortification of crops through the application of Se-containing sprays provides an efficient method to enhance the Se content in the harvested biomass. An optimal candidate for systematic enrichment, guaranteeing a broad trophic impact, must meet several criteria: (i) efficient accumulation of Se without compromising crop yield, (ii) effective conversion of mineral Se fertilizer into usable organically bound Se forms (Se_org_), (iii) acceptance of a Se-enriched crop as livestock feed, and (iv), interest from the food processing industry in utilization of Se-enriched outputs. Hence, priority should be given to high-protein leafy crops, such as soybean.

**Methods:**

A three-year study in the Czech Republic was conducted to investigate the response of field-grown soybean plants to foliar application of Na_2_SeO_4_ solutions (0, 15, 40, and 100 g/ha Se); measured outcomes included crop yield, Se distribution in aboveground biomass, and the chemical speciation of Se in seeds.

**Results and Discussion:**

Seed yield was unaffected by applied SeO_4_^2-^, with Se content reaching levels as high as 16.2 mg/kg. The relationship between SeO_4_^2-^dose and Se content in seeds followed a linear regression model. Notably, the soybeans demonstrated an impressive 73% average recovery of Se in seeds. Selenomethionine was identified as the predominant species of Se in enzymatic hydrolysates of soybean, constituting up to 95% of Se_org_ in seeds. Minor Se species, such as selenocystine, selenite, and selenate, were also detected. The timing of Se spraying influenced both plant SeO_4_^2-^ biotransformation and total content in seeds, emphasizing the critical importance of optimizing the biofortification protocol. Future research should explore the economic viability, long-term ecological sustainability, and the broad nutritional implications of incorporating Se-enriched soybeans into food for humans and animals.

## 1 Introduction

Selenium (Se) is an essential micronutrient in human and animal nutrition, but plants do not need it for relevant metabolic processes. Selenium has an important role in the enzymatic quenching of reactive oxygen species via selenoproteins, which participate in the synthesis of antioxidant enzyme such as glutathione peroxidase (GPx) (Sarwar et al., 2020). Selenium deficiency in the human diet poses a risk of cardiovascular disease and heart attack and has been linked to a higher risk of cancer. It affects the nervous system and may contribute to Alzheimer’s disease, anxiety and depression (Hossain et al., 2021). Recommended minimum daily intake of Se is 55 µg/day and in Europe the daily intake of Se is in the range of 20 to 40 µg/day (Rayman, 2012). Se supplementation, even in apparently Se-replete individuals, has a pronounced effect on the immune system, enhancing the proliferation of activated T cells and increasing lymphocyte-mediated tumour cytotoxicity (Rayman, 2012). According to Zhang et al. (2020) selenate (Se^VI^), selenite (Se^IV^), selenomethionine (SeMet) and selenocysteine (SeCys) are the most important species of Se taken in the diet. Organically bound Se is 85-95% available to the body compared to mineral forms which are only around 40-50% available (Niedzielski et al., 2016).Therefore, it is important to increase the uptake of Se by plants and ultimately the content of Se in the human diet to alleviate human disorders caused by Se deficiency.

Biofortification is the process of enriching crops with essential micronutrients and other health-promoting substances in order to improve the quality of human food or animal feed (Broadley et al., 2006; Schiavon et al., 2020). The consumption of dietary supplements high in Se, including yeast-based supplements, appears to be a safe and effective option for improving human nutrition (Rayman, 2012). However, dietary supplements are relatively expensive, and it is likely that only a small proportion of the population will adopt such personal intervention measures (Broadley et al., 2006).

To increase the Se content of plants, both soil amendments and foliar applications have been tested, the latter method showing significantly higher efficiency. The selenium compounds, Na_2_SeO_4_ and Na_2_SeO_3_, are most commonly used in conventional fertilizers, as they are more economical than organic Se fertilizers (Broadley et al., 2006; Broadley et al., 2010). However, the availability of Se to plants from the soil is highly dependent on physicochemical properties, such as pH and redox potential of soil, chemical speciation, and the activity of soil microorganisms (Lyons, 2010; Mrština et al., 2022). All these soil-related factors render the Se applied fertilizer less plant-available under prevailing field conditions (Schiavon and Vecchia, 2017), thus foliar application of Se salts may be the best option for lowering the risk of Se immobilization in the soil. However, the relatively low threshold of toxicity, the potential negative effects on the availability of other minerals to plants, and the chemical form of Se used in foliar fertilizers must all be considered to avoid negative effects on crop yield (Poblaciones et al., 2014b).

Solutions of both selenite and selenate salts sprayed on leaves have been shown to be safe and effectively absorbed by crops such as rice, wheat and lettuce (Meenakshi et al., 2010), soybean (Silva et al., 2023), chickpea (Poblaciones et al., 2014a), and maize and beans (Ngigi et al., 2019). Selenates and selenites can enter the leaves through the cuticle, stomata, trichomes, stigmata and hydathodes (Wang et al., 2016), and are then transported by the symplastic pathway to the chloroplasts, where they are metabolized to seleno-amino acids through the sulphate pathway (Lidon et al., 2019).

Soybean is one of the most important high protein crops as it contains from 35 to 40% proteins in the seeds. In addition, soybean appears to be an advantageous candidate for biofortification due to its efficient incorporation of Se into selenoproteins (Dai et al., 2020). Soybean protein is used in infant formula, dietary supplements and various food products, and its processed waste is used as a high protein feed for livestock (Yang et al., 2003). Considering the soybean´s leaf morphology and high protein content, it is surprising that there has been only a limited number of studies dealing with soybean biofortification. Therefore, our aims were: (i) to quantify the relationship between the dose of foliar-applied selenate and the Se content in the seeds, for production of soybeans moderately to highly enriched in Se, (ii) to evaluate the biotransformation potential of soybean at high foliar application rates of selenate, and (iii) to identify the main Se species accumulated in the seeds at a high application rate.

## 2 Materials and methods

The three-year field experiment was established in 2020 and continued through 2021 and 2022 in the location of Doudleby nad Orlicí (GPS 50°7´10.89´´N, 16°15´4.595´´E) in East Bohemia, Czech Republic, at an altitude of approximately 273 m above sea level. The soil is characterized as Phaeozem (clay-loam) on loess loam, medium heavy, without skeleton, and with favourable moisture conditions. The content of Se in soil was 0.03 ± 0.005 mg/kg, content of C_tot_ was 4.7% and content of N_min_, 4.39 mg/kg, and available P and K (Mehlich III) was 21 and 184 mg/kg, respectively.

**Figure 1:**
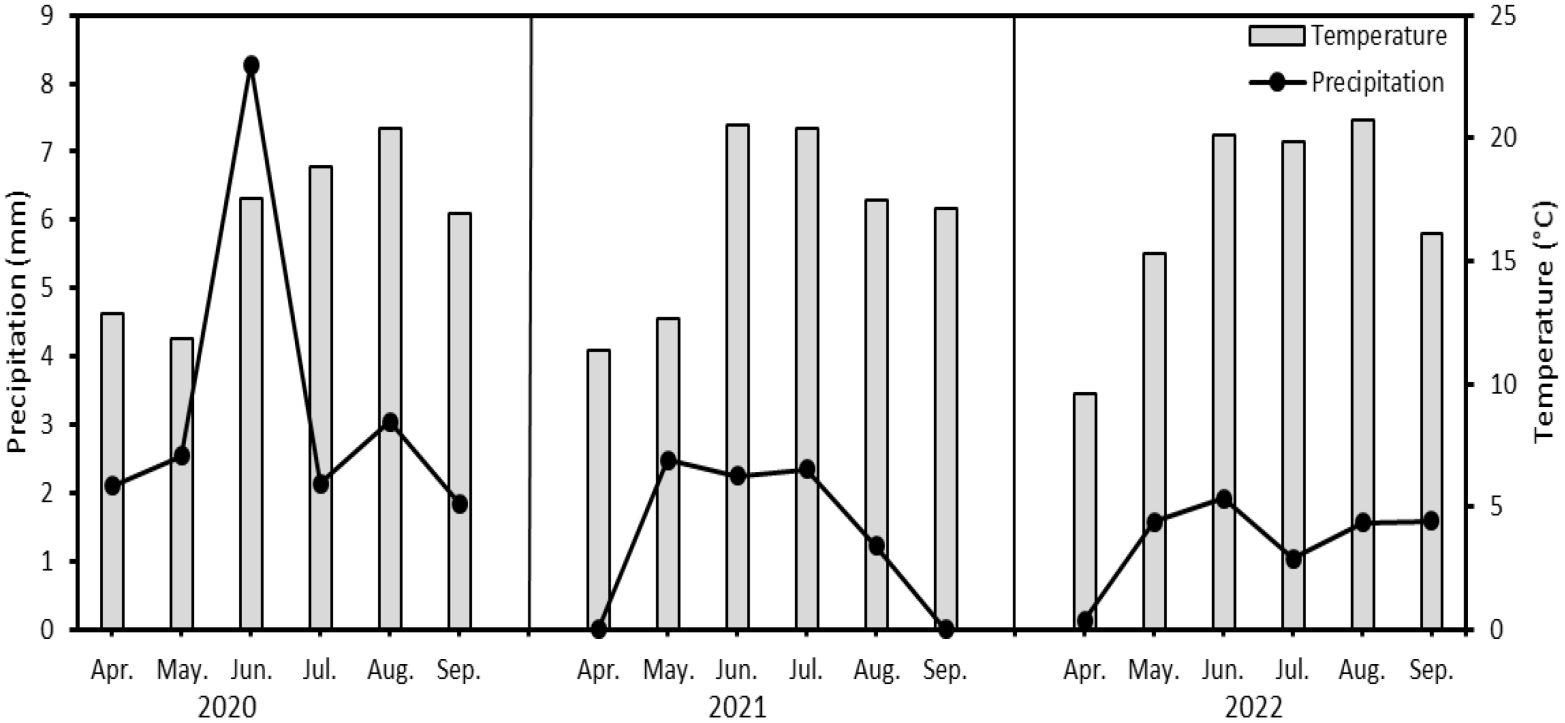
Average temperature and precipitation (monthly aggregated data) during vegetative growth of soybean (*Glycine max* L.) in years 2020-2022.

Conventional soil and plant management was chosen for tillage and sowing. Basic fertilization before sowing was carried out with NPK 15-15-15 at a dose of 100 kg/ha. The soybean variety ‘Saatbau Bettina’ was sown at the end of April and treated against weeds, diseases and pests during the growing season. The weather patterns during the individual growing seasons are shown in **Figure 1**, and the data used were from a meteorological station 2 km from the crop field.

The experiment was set up in four treatment plots, each with an area of 25 m^2^ (5×5 m). The first treatment was the control, treated with drinking water only at the time of Se application. The selenate-treated plots received 15, 40 or 100 g Se/ha at BBCH 60 (first flowers to bloom). In the last year (2022), another phenological phase BBCH 20 (first secondary shoot visible) was included in the experimental design. The weather on the day of application was windless, and the spraying was done in the morning or evening hours when there was no risk of sunburn and no precipitation for at least 24 hours after application. Sodium selenate (Sigma Aldrich, Germany) was applied using a manual backpack sprayer. The total volume of the Se solution was 5 L per plot. The crops were rotated each year. Winter wheat (*Triticum aestivum* L.) was always planted as the pre-crop. The plants were harvested in mid-September. Four 1 m^2^ subplots were harvested from each treatment area. Fresh plants were weighed and then dried at 35°C. Dry soybean plants were separated into seeds and straw and weighed again.

### 2.1 Determination of total Se content in soybean plants

The soybean samples except the seed were homogenized using a 1 mm mesh grinder (MF 10 basic, IKA, Germany). Soybean seeds were ground in a mortar and pestle and passed through a 0.5 mm mesh sieve. The biomass (400 mg) was digested with a mixture of 65% HNO_3_ (8 mL) and 30% H_2_O_2_ (2 mL) at 190°C using a closed-vessel microwave system (Ethos 1, MLS GmbH, Germany). Several aliquots of the seed powder (<0.5 mm) were defatted as follows: 4 g samples were weighed into 50-mL polypropylene tubes and 20 mL of n-hexane was added. The samples were shaken on a reciprocal shaker at 150 rpm for 60 min, centrifuged at 740×g for 5 min, and the hexane layer was decanted. The extraction was repeated three times until the upper layer became clear. The residual extractant was allowed to vaporize out of the tubes in a fume hood for 24 h. The seed powder was digested as described above. Determination of total Se in seeds and straw was made from natural ground samples and Se for speciation analyses from defatted soybean samples in water-diluted (≥ 18.2 MΩ/cm, Millipore, SAS, France) digests was performed by inductively coupled plasma mass spectrometry (ICP-MS; Agilent 7700x Agilent Technologies Inc., USA) operated in He mode. Two certified reference materials (CRMs) were included in the procedure for quality assurance, namely tomato leaves (NIST,SRM 1573a) and bovine liver (BCR-185R).

### 2.2 Selenium speciation analysis

Samples of defatted seed powder (200 mg) were weighed into 15-mL polypropylene tubes and pre-incubated with 5 mL of 30 mM Tris-HCl buffer (pH 7.25) in an ultrasonic bath at 38 ± 2°C for 30 min. Afterwards, the samples were mixed with 1 mL of a solution containing protease XIV from *Streptomyces griseus (*10 mg/mL), and 1 mL of a solution containing protease XXIII from *Aspergillus melleus (*10 mg/mL). Both enzymes were purchased from Sigma Aldrich (Germany) and dissolved in 30 mM Tris-HCl buffer prior to use. After homogenization on a vortex mixer (5 s), the samples were placed in the ultrasonic bath and incubated for 120 min under the same conditions. The tubes were then shaken on a rotator (30 rpm) for 30 min, centrifuged (2690×g) for 5 min and filtered through a syringe filter (0.22 µm, cellulose acetate). After dilution of the filtrate with Milli-Q water or a mobile phase (see below) as appropriate, two aliquots were obtained, one for determination of total Se extraction efficiency and the second for speciation analysis of Se by a chromatography-mass spectrometry technique (HPLC-ICP-MS). A high-performance liquid chromatography (HPLC) method, more specifically a reversed-phase ion-pair chromatography, was performed on an Agilent 1260 (Agilent Technologies Inc., USA) equipped with a C18 Pyramid column (250 × 4.6 mm, 5 µm; Macherey-Nagel, Germany). An isocratic elution system was used to separate five individual species [selenocystine (SeCys_2_), methylselenocysteine (MeSeCys), selenomethionine (SeMet), selenite (Se^IV^), and selenate (Se^VI^)]. The measurement conditions and instrumental parameters followed Praus et al. (2019), The mobile phase contained 20 mM ammonium acetate, 12 mM (NH_4_)_2_SO_4_, 1 mM tetrabutyl ammonium hydroxide (TBAH), and 2.5% (v/v) methanol, pH 5.2. The coupled instruments were calibrated in the range of 0.3-90 ug/L Se for each Se species considered.

### 2.3 Calculations and statistical analyses

Se recovery was calculated using total Se measurements, as follows:


$$\text{Se recovery }\left[\%\right]=\;\frac{\left(\text{Yield }\left[\frac{t}{ha}\right]\times {\text{ Se}}_{treatment}\;\left[\frac{g}{t}\right]\right)–\left(\text{Yield }\left[\frac{t}{ha}\right]\times {\text{ Se}}_{control}\;\left[\frac{g}{t}\right]\right)}{{\text{Se}}_{dose}\;\left[g/ha\right]}\times 100$$

Statistical evaluation of the yield and content of total Se was made on repetitions of n = 4, and Se species from two repetitions (n = 2). Analysis of variance (ANOVA), Tukey’s HSD test and regression analysis at a significance level of *p* ≤ 0.05 were performed using Statistica 12 software (Statsoft, USA).

## 3 Results

### 3.1 Yield of soybean biomass

**Figure 2:**
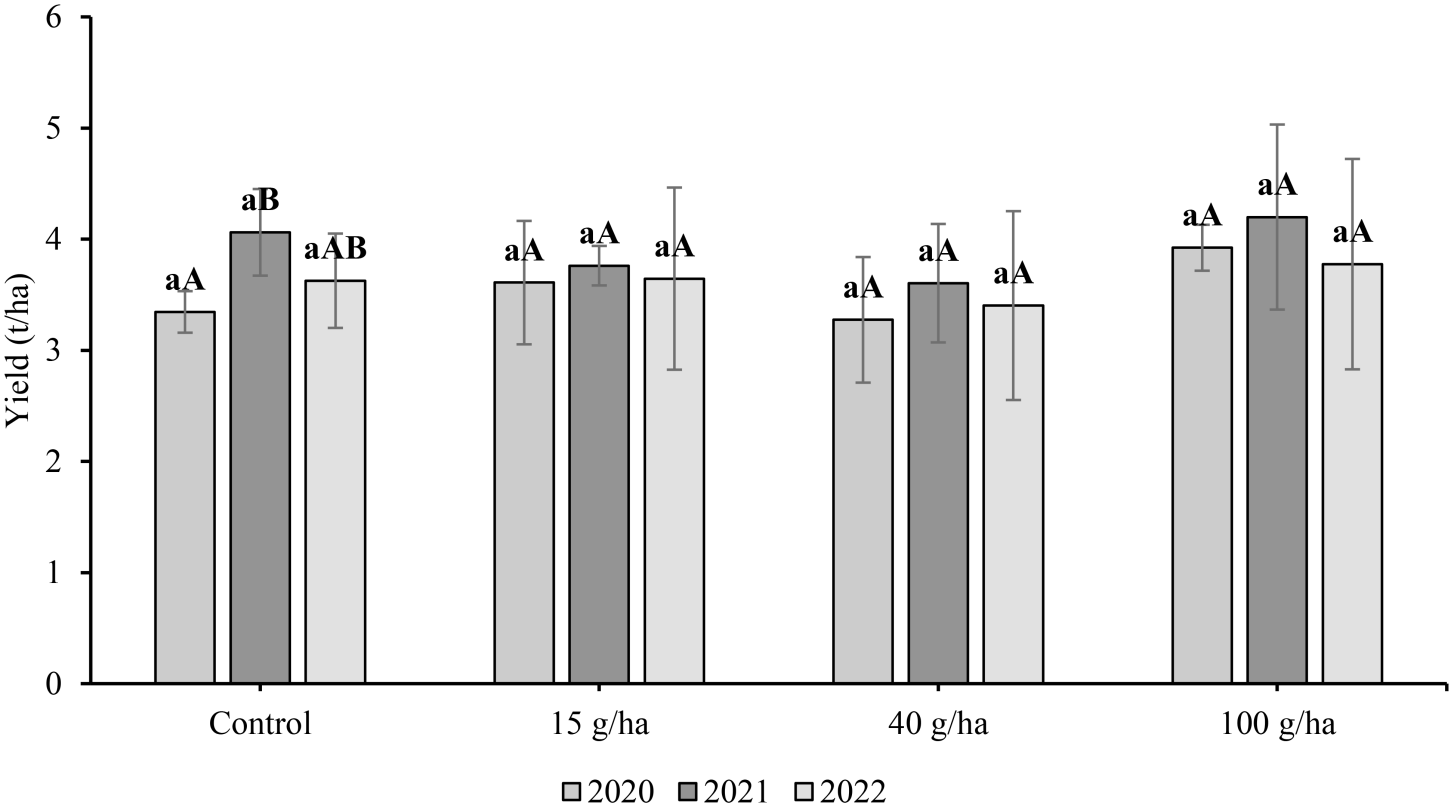
Comparison of soybean (*Glycine max* L) seed yield (t/ha) harvested in 2020-2022. Different lowercase letters in the columns indicate a statistically significant difference between the treatments in a given year and uppercase letters in the columns indicate statistically significant differences over the years in one treatment according to one-way analysis of variance (*p*<0.05), n = 4.

In this three-year experiment we examined the effect of foliar selenate application on soybean seed yield (**Figure 2**). The highest seed yields for a particular treatment were recorded in 2021. However, the influence of growing season was only statistically significant (*p*<0.05) for the control treatment. The highest seed yields were obtained after the highest rate of Se application (100 g/ha), namely 3.92 t/ha (2020), 4.20 t/ha (2021) and 3.78 t/ha (2022). However, the applied Se dose had no significant effect on seed yield (*p*>0.05).

**Figure 3:**
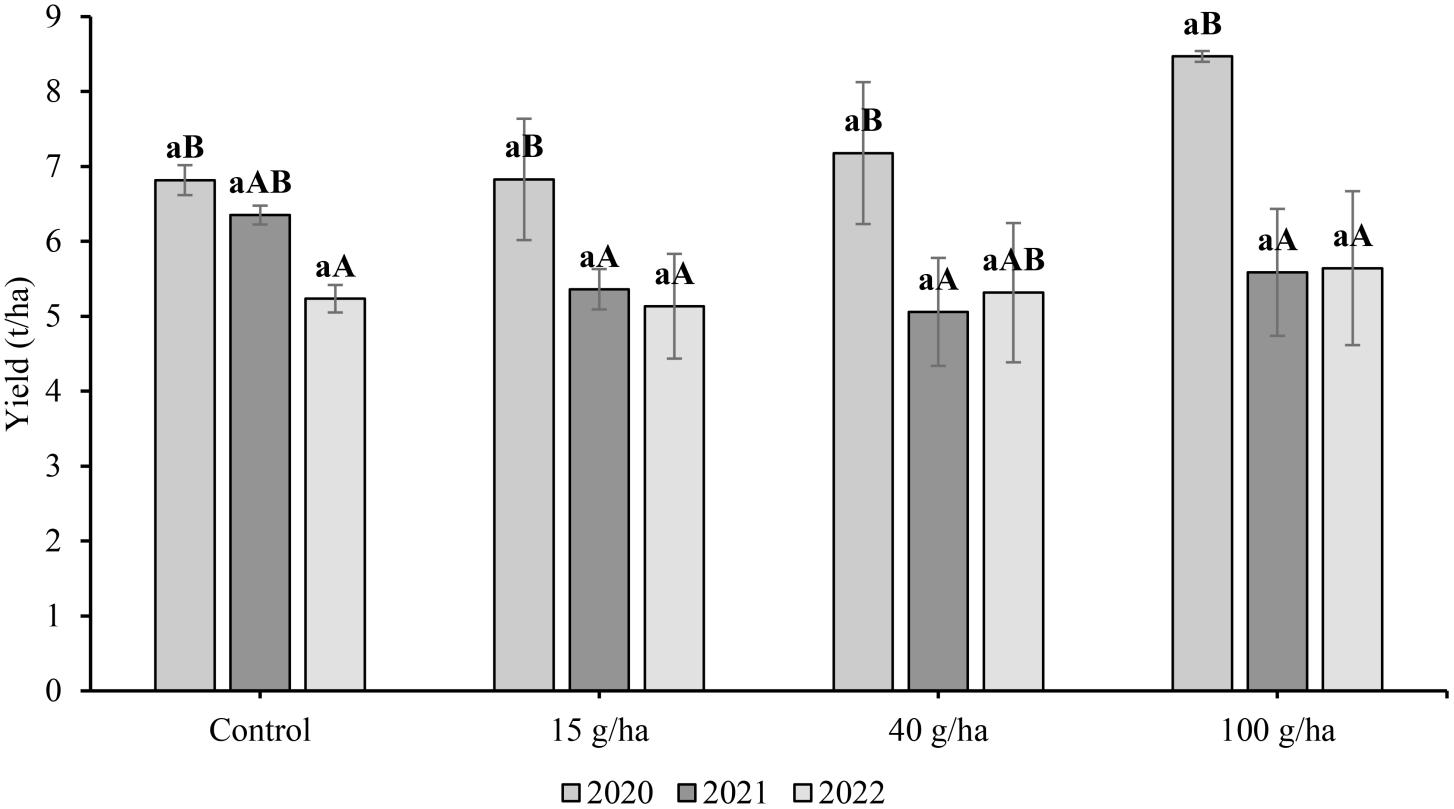
Comparison of soybean (*Glycine max* L.) straw yield (t/ha) harvested in 2020-2022. Different lowercase letters in the columns indicate a statistically significant differencebetween the treatments in a given year and uppercase letters in the columns indicate statistically significant differences over the years in one treatment according to one-way analysis of variance (p<0.05), n = 4.

The yields of straw were also measured and the differences between them are shown in **Figure 3**. The highest straw yields for a particular treatment were found in 2020. This was due to the greater rainfall in June (approx. 8 mm) and hence better straw growth than in other years. The influence of growing season on straw yield was significant (*p*<0.05). The highest mean straw yield (8.47 t/ha) was for the 100 g/ha treatment in 2020 and the lowest yield (5.64 t/ha) was in 2022. In 2021, the highest yield was recorded for the control treatment (6.35 t/ha). The effect of applied Se dose on straw yield was not statistically significant (*p*>0.05).

### 3.2 Content of total selenium in seeds and straw and selenium recovery

**Figure 4:**
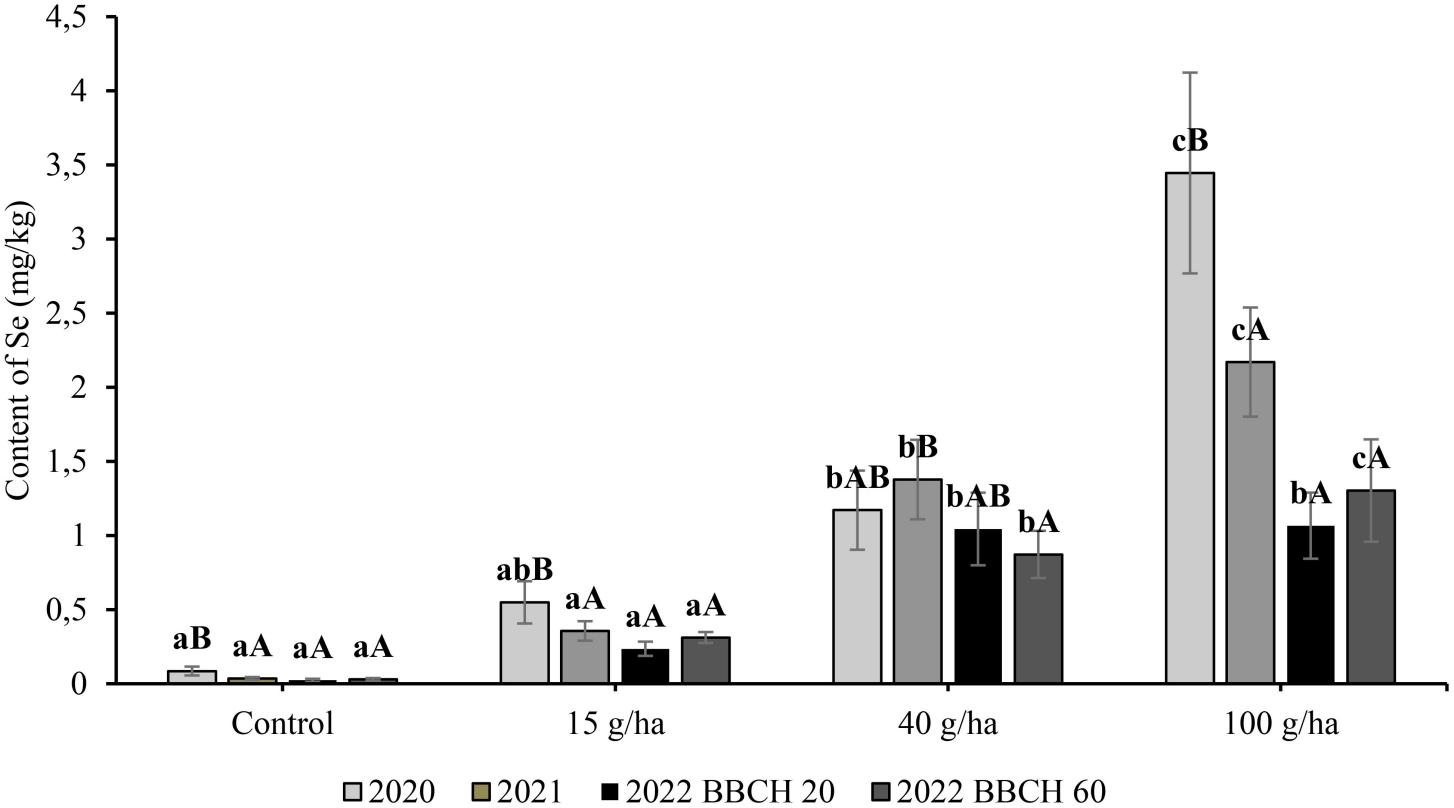
Content of total selenium in straw (b) in soybean (*Glycine max* L.) in 2020-2022. Different lowercase letters in the columns indicate a statistically significant difference between the treatments in a given year and uppercase letters in the columns indicate statistically significant differences over the years in one treatment according to one-way analysis of variance (p<0.05), n = 4.

**Figure 5:**
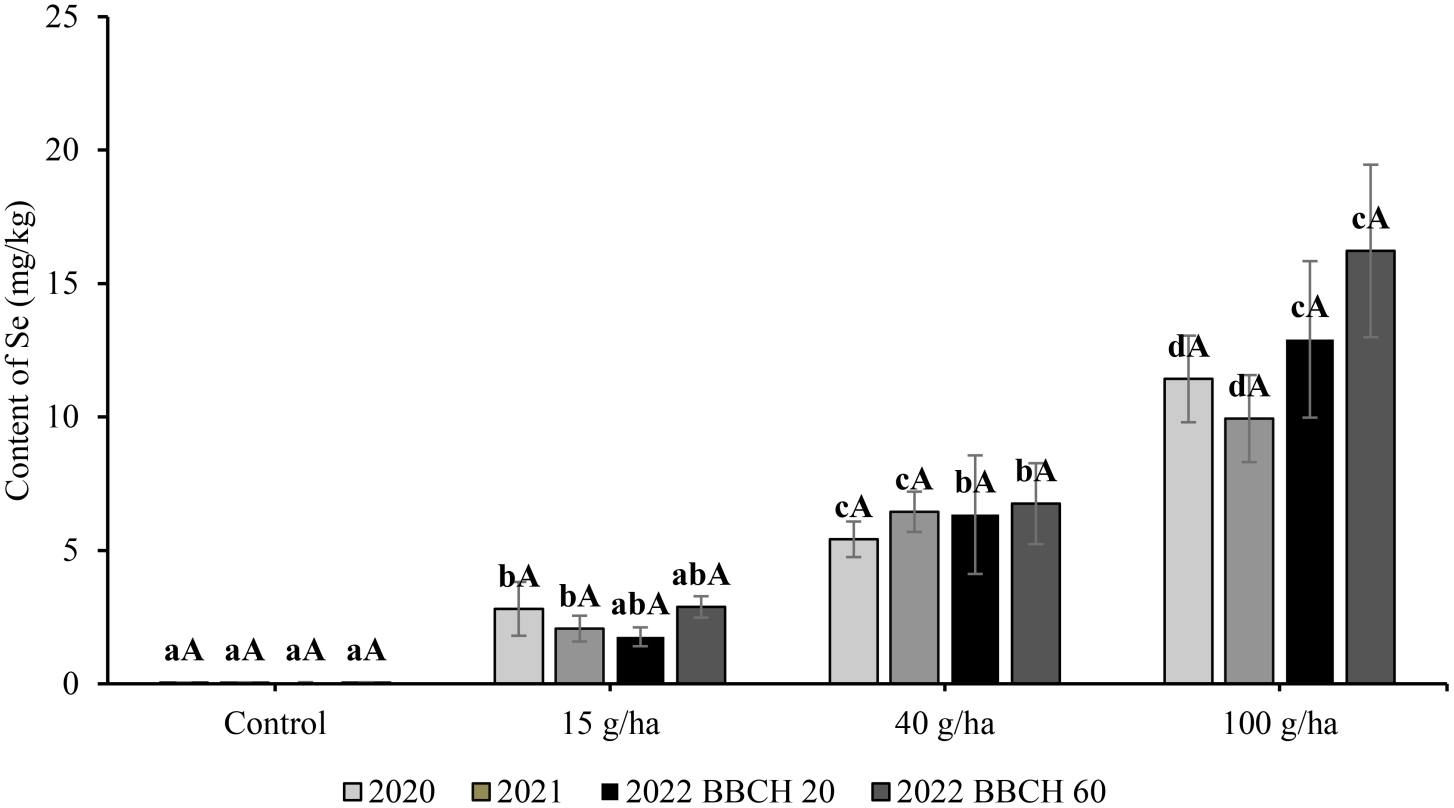
Content of total selenium in seeds in soybean (*Glycine max* L.) in 2020-2022. Different lowercase letters in the columns indicate a statistically significant difference between the treatments in a given year and different uppercase letters in the columns indicate statistically significant differences over the years in one treatment according to one-way analysis of variance (p<0.05), n = 4.

In the three-year experiment, Se content in the seeds in the control treatment without Se application remained stable in the range of 0.04 to 0.09 mg/kg. The content of Se in seeds was significantly (*p*<0.05) affected by the applied selenate dose. The relationship showed a very good fit by linear regression (**Figure 4**), where the y-axis is content of Se, and the x-axis shows applied dose (*a* = slope). The highest Se content in the seeds was recorded at the highest application rate (100 g/ha) (**Figure 5**), and the greatest influence of growing season over the three consecutive years (9.94-16.22 mg/kg Se) also occurred with that variable. The highest Se contents in the seed for a particular treatment were recorded in 2022, although the exceptionality of this season was evaluated as statistically significant only for the application of 100 g/ha Se. The selenium content of seeds in 2022 was 1.76, 6.34 and 12.91 mg/kg at BBCH 20 and 2.88, 6.75 and 16.22 mg/kg at BBCH 60 for the 15, 40 and 100 g/ha Se treatments, respectively.

In comparison to the slopes of the increase in Se content in seeds (0.10-0.16), the slope of the enrichment for straw was flat (**Figure 4**), which was especially evident in 2022 (0.01). The highest Se content in straw was 3.45 mg/kg after an application of 100 g/ha Se in 2020. At the same application rate, Se content in the following years was significantly lower at 2.17 mg/kg (2021) and 1.30 mg/kg (2022). Compared with the Se content in seed, the influence of growing season on Se content in straw was also more frequent in other treatments. The year 2022 was characterised by the lowest Se content in straw for a particular treatment. The selenium content of straw in 2022 was 0.24, 1.04 and 1.07 mg/kg at BBCH 20 and 0.31, 0.87 and 1.30 mg/kg at BBCH 60 for the 15, 40 and 100 g/ha Se rates, respectively. The regression of Se content in seeds on the applied Se dose was linear and the coefficients of determination (R^2^) varied from 0,93 to 0,99.

In the aboveground biomass the average total Se recovery from 2020-2022 was 78%, 70% and 65% for 15g/ha, 40 g/ha and 100 g/ha Se, respectively. Thus, the efficiency decreased with increasing Se rate so that the highest efficiency was achieved with the lowest Se dose.

**Figure 6:**
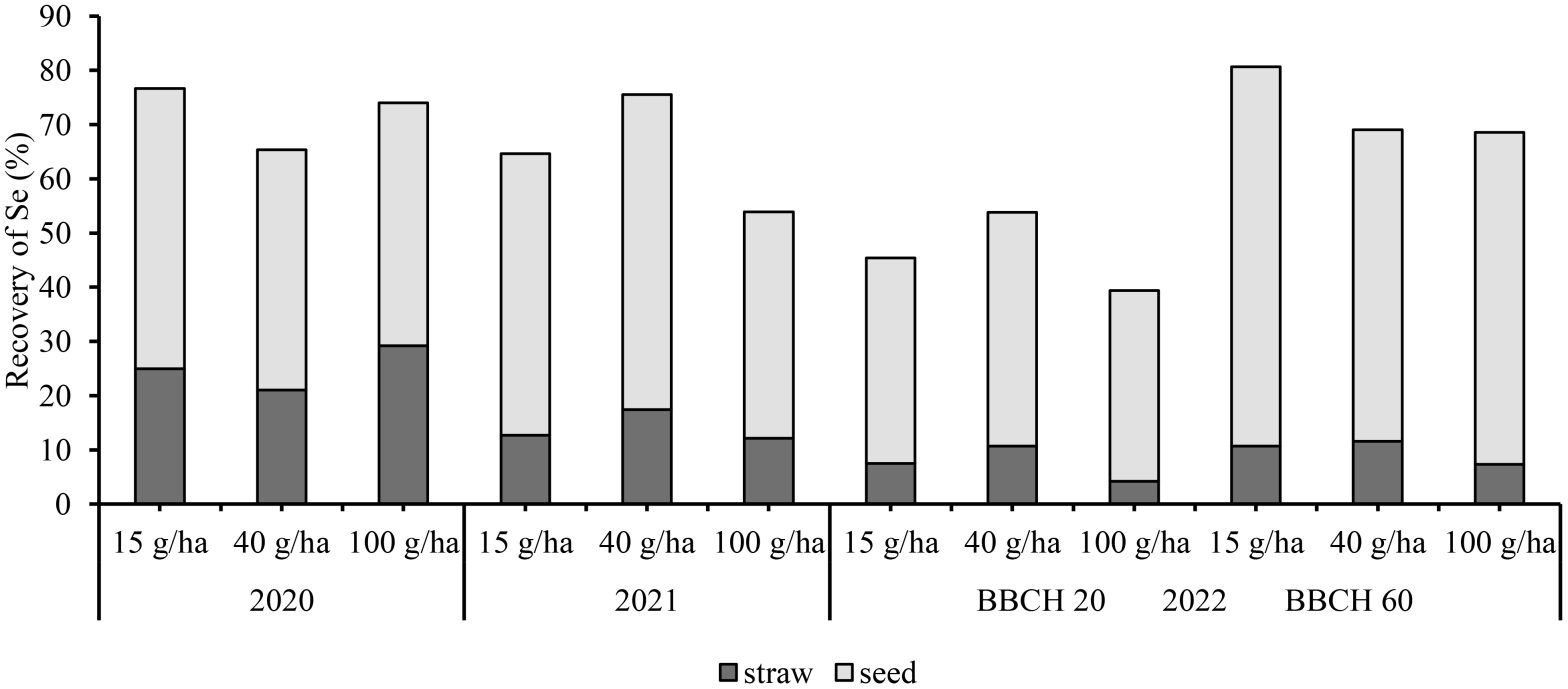
Recovery of Se in soybean (seed + straw).

The recovery of Se for all treatments in the earlier phenological phase (BBCH 20) was lower at an average of 46% compared with the later phase (BBCH 60), with an average efficiency of 73% (**Figure 6**). The highest recovery at BBCH 20 was 53% for the 40 g/ha treatment, while at BBCH 60 the recovery was 68% for the same treatment.

### 3.3 Content of Se species

Chemical speciation analysis of Se was performed on the soybean seed samples from 2022. After fat removal, the Se content was shown to increase by an average of 23 ± 5%. The average extraction efficiency was 83 ± 6% and the column recovery was 76 ± 4%. Regardless of the treatment, the main Se species in the enzyme hydrolysate was SeMet (**Table 1**). In the control, the content of SeMet was as low as 0.08 mg/kg Se, but it increased linearly up to 15.69 mg/kg Se, when 100 g/ha Se was applied at BBCH 60. Some minor Se species were also quantified, especially in Se-treated plants, namely selenocystine (SeCys_2_) (0.12-0.43 mg/kg Se), Se^IV^ (0.07-0.61 mg/kg Se) and Se^VI^ (0.03-0.27 mg/kg Se). MetSeCys content was below the limit of detection. Apparently, selenate application rate as well as the phenological phase at the time of application impact the distribution of Se species in the seed and the biotransformation potential of soybean plants converts selenate to organically bound Se.

**Table 1:** Contents of Se species in soybean seeds.

Treatments	Total Se (defatted)	SeCys_2_	MetSeCys	SeMet	Se^IV^	Se^VI^			ΣSe	Extraction efficiency (%)
	(mg/kg)									
Control	0.09^a^	< 0.01	< 0.01	0.08^a^	0.02^a^		< 0.01	0.10^a^		**89**
BBCH 20 - 15 g/ha	3.15^b^	< 0.05	< 0.05	1.73^b^	< 0.05		< 0.05	1.73^b^		**61**
BBCH 60 - 15 g/ha	3.66^b^	< 0.05	< 0.05	2.45^b^	0.07^b^		0.06^b^	2.58^c^		**67**
BBCH 20 - 40 g/ha	7.06^c^	0.14^a^	< 0.05	4.78^c^	0.19^c^		< 0.05	5.11^d^		**70**
BBCH 60 - 40 g/ha	11.95^d^	0.20^b^	< 0.05	7.19^e^	0.27^d^		0.19^c^	7.85^e^		**62**
BBCH 20 - 100 g/ha	8.08^e^	0.12^a^	< 0.10	5.91^d^	0.27^d^		< 0.10	6.29^f^		**75**
BBCH 60 - 100 g/ha	23.79^f^	0.43^c^	< 0.10	15.69^f^	0.61^e^		0.27^d^	16.22^g^		**68**

Different lowercase letters in the columns indicate a statistically significant difference between the treatments in one parameter according to one-way analysis of variance (p<0.05), n = 2.

The bold values indicate the percent extraction efficiency, which indicates what percentage of Se specie was extracted from the sample.

## 4 Discussion

Many researchers have tested the hypothesis that Se application can increase crop yield, based on the involvement of Se compounds in reducing the level of oxidative stress in plants. To the best of our knowledge, however, the majority of reports in the literature showed no effect of exogenous Se on yield (Broadley et al., 2010; Ducsay et al., 2016). These results are in line with our study, as well as those of Yang et al. (2003) who also observed no improvement in soybean seed yield with Se application rates as high as 200 g/ha, but did record higher yields than in our study. One possible explanation was their higher soil Se content (0.296 mg/kg). On the other hand, the study of Nawaz et al. (2015) claims that Se had a negative effect on yield. Astaneh et al. (2019) reported that the application of Se to increase the yield of garlic (*Alium sativum* L.) increased salinity resistance and reduced oxidative stress by increasing the activity of antioxidant enzymes. Also, Yildiztugay et al. (2017) confirmed the reduction of ROS under drought stress by Se application to maize (*Zea mays* L.).

**Figure 7:**
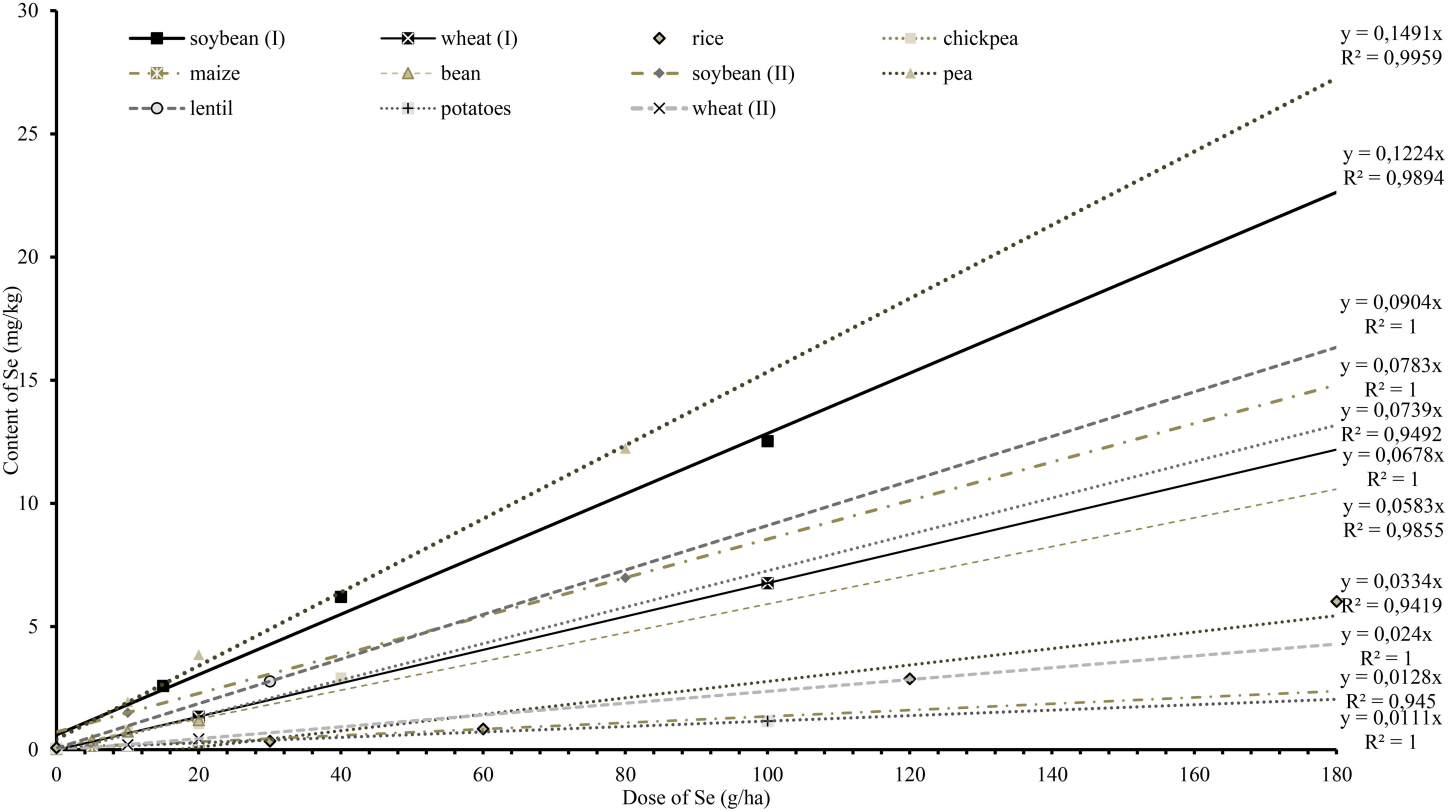
Regression curve slopes modelling the relationship between applied Se dose and Se content in crops, based on results from our study and literature data: pea, a (slope) = 0.1491 (Poblaciones et al., 2013); soybean (I), a = 0.1224; lentil, a = 0.0904 (Rahman et al., 2015); soybean (II), a = 0.0783 (Silva et al., 2023); chickpea, a = 0.0739 (Poblaciones et al., 2014a); wheat (I), a = 0.678 (Wang et al., 2020); bean, a = 0.0583 (Ngigi et al., 2019); rice, a = 0.0334 (Lidon et al., 2019); wheat (II), a = 0.024 (Ducsay et al., 2016); maize, a = 0.0128 (Ngigi et al., 2019); potatoes, a = 0.0111 (Zhang et al., 2019).

High uptake efficiency and linear dose responses are required characteristics for successful biofortification of crops by foliar application, allowing the achievement of a desired level of Se in the edible parts. In general, high-protein crops are considered suitable candidates for agronomic selenium biofortification because they incorporate Se instead of sulphur in the protein. Silva et al. (2023) measured a Se content of 1.64 mg/kg in soybean, after they applied 10 g/ha Se. Our study recorded that in 2020, 2021 and 2022, an average of 12.53 mg/kg Se was measured in soybean seeds at an applied rate of 100 g/ha Se. Similar results were achieved by Silva et al. (2023) who used foliar application of sodium selenate at 80 g/ha Se to the soybean genotypes Lanca and M5817 and measured 7.01 mg/kg and 7.73 mg/kg Se in seeds, respectively. To allow comparison among different studies focused on crop biofortification, we performed linear regression analysis to obtain the slope characterizing the crop response (the content of Se in edible product) to foliar Se (**Figure 7**). For high-protein crops, graphs of Se content vs application rate in the literature had slopes ranging from y = 0.0583x for beans to y = 1.491x for peas. In terms of growth phase comparisons, in the earlier phase selenium accumulated more in the straw (stems, leaves), while in the generative phase the plants accumulated mobile selenate more in the seeds. This observation was supported by Terry et al. (2000), who concluded that applied forms of Se were incorporated into biomass at earlier stages and into generative organs at later stages.

Results of biofortification studies of starch crops did not show significant Se accumulation in grains or tubers. Ducsay et al. (2016) reported that foliar sodium selenate increased the total selenium content in the seeds of winter wheat (*Triticum aestivum* L.), and regression of their data gave a slope of y = 0.0334x. Also, Ngigi et al. (2019) found that maize (*Zea mays* L.) accumulated 0.31 mg/kg at a Se dose of 20 g/ha (y = 0.0128x). Lastly, Zhang et al. (2019) applied foliar selenium to potatoes (*Solanum tuberosum* L.) and measured a content of 1.16 mg/kg (y = 0.0111x) in tubers. For starchy crops, Se content ranged from y = 0.0111x, in potatoes to y = 0.678x in wheat (Sarwar et al., 2020), and from this, we concluded that crops with a higher percentage of carbohydrates and lower percentage of protein were less suitable for Se biofortification. Although the maximum Se content in livestock feed is limited by the EU to 0.568 mg/kg DW in feed (Regulation-EC, 2003), it may be advantageous to produce highly enriched material of plant origin, such as high-Se protein concentrates, for supplementation purposes or further industry processing. Although practically all crops respond to increasing Se doses with a linear increase of Se content in biomass, the efficiency of high-Se biomass production is highly variable for different crops (**Figure 7**).

The efficiency and economic viability of the application of biofortification to high-protein farm crops hinges upon the linear scalability of Se content in biomass, the high recovery of Se facilitated by generative plant parts, and the year-to-year stability of these processes. With regard to Se recovery in aboveground biomass, a rate of 15 g/ha (78%) seems to be the best choice in terms of balancing efficiency with economy. Se recovery in soybean plants was 39.5% and 34.3% at an application rate of 10 g/ha and 80 g/ha Se, respectively (Silva et al., 2023). On the other hand, Deliboran (2023) found higher recoveries in grain maize at 49.9% and 43.93% for Se applied at a rate of 15 g/ha and 100 g/ha, respectively. In our study, the recovery of Se from soybean was always higher at low application rates than high rates, and this finding was corroborated by other authors. Concerning the efficiency of biofortification, the seasonal variations in 2020 influenced selenium accumulation in straw. Conversely, in 2022, there was an observed rise in selenium transport to the seeds. There was a decreasing trend with higher application rates in the coefficient of variation (CV) in soybean seeds: 24% for the 15 g/ha treatment, 15% for the 40 g/ha, and 18% for the 100 g/ha treatment. The opposite trend was observed for soybean straw when the coefficient of variation increased from 12%, to 18%, and then to 26%, with increasing Se application rates. These differences in Se content trends were probably due to the lower accumulation capacity of seeds, where Se had to be transported, compared to the leaves, which were in direct contact with the spray and could have incorporated higher amounts of applied Se.

The efficiency of absorbed inorganic Se species to be converted into organic Se species by plants is another important parameter for consideration. Due to incomplete extraction of Se from the biomass, the suboptimal degree of protein hydrolysis, other technique-specific issues, such as incomplete chromatographic column recovery and the presence of unidentified Se species (including species below detection capability of the technique) in plant extracts, the task of comparing individual studies and fortified plants becomes challenging. Nevertheless, the degree of Se biotransformation appears to be higher in high-protein crops. Di et al. (2023) enhanced the selenium content in wheat plants through foliar application, employing doses of 15 g/ha and 30 g/ha Se^IV^ or Se^VI^. Their analysis of enzymatic grain extracts revealed a substantial proportion of organic Se species, ranging from 93-97%. It should be noted that these percentages were not adjusted for the extraction efficiency, which ranged from 62-80%. Similarly, Wang et al. (2022) measured an extraction efficiency of 87-96% for wheat grain after foliar application of selenate. A conversion of ≥80% of total Se to organic species in grains was reported for selenate-sprayed (75 g/ha Se) rice plants (Deng et al., 2017) and selenate-sprayed (10 g/ha and 80 g/ha Se) soybean plants (Silva et al., 2023). Even low-protein starchy crops such as potato may exhibit a high conversion efficiency, although Zhang et al. (2019) demonstrated the importance of the type of Se species applied. Consequently, they found only 1.5% residual inorganic Se species in tubers after spraying Se^IV^, as compared with up to 31.9% in the case of Se^VI^ application. It may be hypothesized that the biotransformation capacity was exceeded for Se^VI^ at 100 g/ha, because the first reduction step Se^VI^→Se^IV^ is essential for selenate biotransformation. In addition to Se dose, the optimal phenological phase at time of Se application must also be considered. Wheat grains contained only 3.6 ± 1.1% residual Se^VI^ after spraying selenate solution (100 g/ha Se) at the mid-booting stage, while it increased to 11.1 ± 0.1% when applied later at the grain-filling stage (Galinha et al., 2014). Deng et al. (2017) delayed the foliar application of 75 g/ha Se^VI^ to rice from the late tillering to the full heading stage and the content of organic Se species decreased from 88.6% to 80.4% of total Se content. However, spraying at full heading resulted in the Se content of the grain being 2.9-3.5-fold higher compared to that at tillering, implying that achievement of a high Se accumulation in seeds represents a more crucial objective than optimization for the lowest possible residual inorganic Se species. Silva et al. (2023) also detected minor Se species in soybean seeds, some of which were especially low in proportion to SeMet after application of 80 g/ha Se. In addition to Se^IV^ and Se^VI^ species, they reported minor concentrations of SeCys (perhaps detected in the form of its dimer SeCys_2_) and MetSeCys. In our case, SeCys_2_ was quantified up to 0.43 mg/kg Se in seeds showing an increase with growing selenate application rate. However, it is unclear whether the SeCys (SeCys_2_) originated as a central intermediate in the Se metabolic pathway or as a product of protein hydrolysis analogous to SeMet (Sors et al., 2005). The traces of MetSeCys in seeds should be interpreted with caution as this seleno-aminoacid is non-proteinogenic, and thus could be lost during hexane extraction. The major species of Se is SeMet, which is non-specifically incorporated into selenoproteins. In our research, up to 95% of organic selenium amino acids were found in all spray applications in hydrolysed soybean seeds. In the 100 g/ha Se treatment SeMet was present at almost 91%. Silva et al. (2023) measured up to 94.1% SeMet in soybean seeds. Also, Jiang et al. (2018) found SeMet as the most abundant organic selenium compound in hydrolysates of buckwheat seeds to which sodium selenate was applied. We have observed that the total amount of inorganic Se increased with increasing Se dose. However, there was no observable decline in biotransformation efficiency as the doses of Se increased within the tested range.

## 5 Conclusions

In this three-year field experiment, soybean was found to be a good candidate for biofortification, able to accumulate up to 16.22 mg/kg of Se in the seeds with no obvious negative impact on yield (or quality). This enrichment could lead to a reduction of Se deficiency in the diet of livestock and populations. Soybean is able to convert almost 95% of the mineral forms of selenium into organic forms, even at a rate of 100 g/ha Se. The main specie represented is selenomethionine, which is much better absorbed and utilized than the mineral selenate or even selenite. Regression analysis confirmed very close relationships between accumulated Se and applied Se dose in selenium fortified soybeans. The crop was also able to accumulate Se into the straw, which can be used in many ways, as livestock feed, a component of compost or ploughed into the field to enrich the topsoil with selenium.

## Data availability statement

The original contributions presented in the study are included in the article/**Supplementary Material**. Further inquiries can be directed to the corresponding author.

## Author contributions

TM: Data curation, Investigation, Methodology, Visualization, Writing – original draft. LP: Formal analysis, Methodology, Writing – review & editing. JS: Supervision, Writing – review & editing. LK: Investigation, Writing – review & editing. PT: Supervision, Writing – review & editing.
