# Supplementary material for: Foliar selenium biofortification of soybean: the potential for transformation of mineral selenium into organic forms
Source: Front Plant Sci. 2024 May 2;15:1379877. doi: 10.3389/fpls.2024.1379877 (PMC11096529; doi:10.3389/fpls.2024.1379877)
Supplement: Supplementary file 1 [file Table_1.docx]

Supplementary Material

**Suppl. Figure 1.** Regression analysis of content of Se in seeds with different applications of Se.
